# Spinal Cord Injury and Osteoporosis: Causes, Mechanisms, and Rehabilitation Strategies

**Published:** 2013-04-23

**Authors:** Can Ozan Tan, Ricardo A Battaglino, Leslie R Morse

**Affiliations:** 1 Department of Physical Medicine and Rehabilitation, Harvard Medical School, Boston, MA, USA; 2 Spaulding Rehabilitation Hospital, Boston, MA, USA; 3 Department of Mineralized Tissue Biology, The Forsyth Institute, Cambridge, MA, USA; 4 Department of Oral Medicine, Infection, and Immunity, Harvard School of Dental Medicine, Boston, MA, USA

**Keywords:** Spinal cord injury, SCI-related osteoporosis, Chronic post-injury phase

## Abstract

Spinal cord injury (SCI) has a huge impact on the individual, society and the economy. Though advances in acute care resulted in greatly reduced co-morbidities, there has been much less progress preventing long-term sequelae of SCI. Among the long-term consequences of SCI is bone loss (osteoporosis) due to the mechanical unloading of the paralyzed limbs and vascular dysfunction below the level of injury. Though osteoporosis may be partially prevented via pharmacologic interventions during the acute post-injury phase, there are no clinical guidelines to treat osteoporosis during the chronic phase. Thus there is need for scientific advances to improve the rehabilitative approaches to SCI-related osteoporosis. Recent advances in application of a new technology, functional electrical stimulation, provide a new and exciting opportunity to improve bone metabolism and to provide mechanical strain to the paralyzed lower limbs sufficient to stimulate new bone formation in individuals with SCI. The purpose of this minireview is to delineate our current understanding of SCI-related osteoporosis and to highlight recent literature towards its prevention and treatment.

There are approximately six million people living with spinal cord injury (SCI)-related paralysis in the United States – nearly one in every 50 people. Though advances in acute care resulted in greatly reduced co-morbidities in the initial few years following a spinal cord injury, there has been much less progress preventing medical complications associated with SCI in the long-term. Therefore, understanding the long-term consequences of SCI is critical to develop evidence-based rehabilitation programs that would provide optimal treatment for the reversal of co-morbidities.

Individuals with SCI are at increased risk for developing an array of inactivity-related health problems during the chronic stages of injury. Among several consequences of SCI is bone loss (osteoporosis) following injury, which is both rapid in onset and severe in nature. In motor complete SCI, the long bones of the lower extremity adapt to minimal mechanical strain by atrophying. Bone loss occurs rapidly in the acute phase of the injury and slows two to three years after injury [1]. While the nature and magnitude of the effects of SCI on bone vary by skeletal site, sex, and age [2], all individuals with motor complete SCI develop osteoporosis below the level of the injury [1,3,4]. Perhaps as a result, individuals with complete SCI are twice as likely to experience fractures compared to healthy controls [5], and as many as 40% of the individuals with chronic SCI experience fractures [5–8], with the most common occurrence at the metaphyses of the proximal tibia and distal femur [9]. Fractures are discovered after minimal trauma and are most commonly treated with prolonged bed-rest and bracing in many cases. However, the combination of the injury and extended bracing results in prolonged immobility, worsening disability, and serious medical complications, such as pressure ulcer formation, increased pain and spasticity, and lower extremity amputation. Thus, it is critical to develop rehabilitation programs that may effectively reverse the sequelae of prolonged lower extremity disuse and minimize the medical complications due to osteoporosis secondary to lower extremity paralysis. Unfortunately, however, physical therapy does not appear to have proven efficacy [10], and there are no studies that conclusively showed an effective pharmacologic intervention for prevention and treatment of osteoporosis in chronic SCI [11]. Part of the culprit may be that only a small number of individuals with SCI volunteer for long-term studies, and adequately matching individuals by the level, completes, and duration of lesion, as well as age is not always possible [7,11]. Thus long-term longitudinal randomized investigations on osteoporosis in individuals with SCI have been difficult. Nevertheless, the lack of effective rehabilitative strategies underlines the importance of an integrated understanding of the factors, both neural and local, that are involved. The purpose of this mini-review is to delineate our current understanding of SCI-related osteoporosis and to highlight recent literature towards its prevention and treatment.

## Neural Denervation, Limb Unloading, and Mechanisms of Bone Loss Following SCI

Trabecular and cortical bone as well as the bone marrow are innervated by sympathetic neural fibers [12–15], and functional noradrenaline and various neuropeptide receptors have been identified on bone cells [16,17]. Thus, sympathetic innervation appears to play an important role in bone function. In fact, experimental sympathetic denervation in animal models results in reduced bone deposition and mineralization and increased bone resorption [18,19], suggesting a potential direct impact of denervation on bone function. In addition to its direct impact, sympathetic denervation may also have an indirect impact on bone metabolism via vascular dysregulation. For example, interruption of sympathetic signaling causes the opening of bone intravenous shunts, leading to venous and capillary vascular stasis [20,21]. Among the consequences of vascular stasis is osteoclast formation due to local hyper-pressure, which may accelerate bone resorption [22]. Therefore, vascular dysfunction below the level of injury may promote and/or facilitate the development of osteoporosis.

In addition to nervous denervation and subsequent vascular alterations, the rapid loss of bone due to any type of prolonged immobilization is also related to limb unloading and consequent alterations in calciotropic hormones and local messenger systems [23]. The general consensus is that SCI-related bone loss occurs in 2 phases: 1) a rapid, acute phase characterized by increased bone resorption that plateaus somewhere between 18–24 months post-injury and 2) a chronic phase, characterized by inhibition of bone formation with ongoing bone loss that is more gradual in nature [24–27]. Early studies of circulating levels of bone turnover markers in SCI subjects reported that bone formation is suppressed immediately following SCI [28]. Other reports using animal models of hind limb unloading have described immediate osteocyte and osteoblast apoptosis [29], and an increased osteoclastic bone resorption with reduced bone formation [30]. Thus, mechanical unloading following SCI leads to a rapid increase in bone resorption by osteoclasts and suppresses bone formation by osteoblasts, ultimately leading to bone loss.

The discovery of the role of Wnt signaling pathways in bone homeostasis has radically transformed our understanding of the cellular and molecular mechanisms responsible for the adaptation of bone to unloading [31,32]. While Wnt signaling pathways include a large family of growth factors that participate in various developmental events, these pathways are also implicated in adult homeostatic mechanisms [24,33]. For example, dysfunction of Wnt pathways have been implicated in a variety of degenerative diseases and abnormalities, including those associated with impaired bone homeostasis [34]. Indeed, several studies in rodents have defined the central role of Wnt signaling antagonists in the pathogenesis of disuse osteoporosis. Osteocytes, the cells responsible for mechano-transduction in bone, represent the first cellular response to unloading [35], and release sclerostin, a potent Wnt signaling antagonist [36–38]. Several studies have shown that sclerostin levels are inversely proportional to bone mass and that production of sclerostin by osteocytes is dramatically reduced by mechanical loading [1,37,39]. Thus, mechanical unloading results in up-regulation of sclerostin, which leads to reduced Wnt/β-catenin signaling in osteoblasts and to inhibition of bone formation and growth. Moreover, sclerostin causes up-regulation of RANKL (a key factor that promotes osteoclast differentiation), and down-regulation of osteoprotegerin (a key inhibitor of osteoclast differentiation) expression by osteocytes, which leads to increased osteoclast activity and ultimately to bone resorption [40,41]. Thus, in addition to its anti-anabolic role, sclerostin also appears to have catabolic effects.

Recent work has also shown a positive relation between circulating sclerostin levels and bone density in chronic (>5 years) immobility in humans. Considering the mechanism of sclerostin-induced bone loss in acute SCI, this relation in the chronic phase seems paradoxical at first. However, though sclerostin levels may initially increase after SCI in response to mechanical unloading, in the long-term, circulating sclerostin may serve as a biomarker of osteoporosis severity and not a mediator of ongoing bone loss. Indeed, recent research supports this duality. On one hand, sclerostin levels are greatest in subjects with short-term SCI and decrease significantly over the first 5 years post-injury [42]. On the other hand, in subjects with long-term (>5 years post-injury) SCI, sclerostin levels are positively associated with lower extremity bone density and bone mineral content [42].

## Pharmacologic Strategies Toward Treatment of Osteoporosis Following SCI

Currently there are no clinical guidelines for the prevention or reversal of SCI-related osteoporosis. Traditionally, bisphosphonates have been considered as the most appropriate therapy to prevent bone loss following SCI. Bisphosphonates strongly inhibit bone resorption. Various reports indicate that they provide an effective preventive treatment strategy when initiated within 12 months of the injury [43–46], and early bisphosphonate administration increase ash weight, maximal torque capacity, maximal angle capacity and rigidity of the bone atrophied by immobilization [47]. However, the efficacy of bisphosphonate treatment appears to be limited to only within the acute phase (< 1 year) of injury [48]. This may be related to the fact that though bisphosphonates reduce bone resorption, they have limited effect on bone formation [49]. This is explained by the fact that bisphosphonates reduce coupled bone remodeling because they suppress osteoclastic bone resorption, which is required in order for osteoblastic bone formation to proceed.

The role of sclerostin in the adaptation of bone to unloading during the acute phase of SCI suggests that sclerostin may provide an alternative therapeutic target during the acute phase of injury as a prevention strategy to prevent initial, rapid osteoporosis. The higher sclerostin levels in acute SCI and lower levels in chronic SCI strongly suggest that the time frame for limiting bone resorption is limited. Thus, there may be an optimal time frame - the “therapeutic window” - for targeting sclerostin and preventing bone loss following SCI [6]. Unfortunately, however, there is no longitudinal information that defines the kinetics of bone loss and its relation to circulating sclerostin in the acute phase of SCI (i.e., within the first year), when acute mechanical unloading and most bone loss occurs.

The ongoing discussion suggests that although bone loss in individuals with SCI may be partially prevented via pharmacologic interventions, notably during the acute post-injury phase, current pharmacologic treatments do not appear to be capable of reversing bone demineralization. Thus, currently there is no effective pharmacologic intervention for prevention and treatment of disuse osteoporosis due to SCI, especially after the first year of injury. Perhaps as a consequence, a recent, emerging theme in the literature is the utility of novel, non-pharmacologic paradigms that are specifically designed for individuals with SCI to prevent and reverse the bone loss due to prolonged immobility.

## Physical Exercise and Effective Reversal of Osteoporosis Following SCI

Bone is a dynamic organ that modulates the rate of new bone formation in response to varying levels of physical exercise and mechanical strain, and there is already ample evidence that physical exercise in those with SCI is broadly beneficial to health [50,51], improves quality of life [52], and impacts outcome after SCI [53]. Therefore, it would not come as a surprise that physical exercise can reduce, prevent, and even reverse SCI-related osteoporosis. There is evidence of improved circulation in bone vasculature during muscular work. For example, recent work has shown that resting femoral bone blood flow almost doubles in response to isometric exercise, although the increase in blood flow plateaus with increasing exercise intensities [54]. The blood flow response to muscular work appears to be mediated by a metabolically induced stimulus, rather than neural mechanisms [55]. Thus, physical exercise may promote bone blood flow, alleviate the bone vascular dysfunction due to neural denervation, and facilitate bone metabolism and growth in SCI. Furthermore, mechanical loading of the bone during exercise may reverse the alterations in local Wnt signaling cascade that occur due to immobilization and unloading, contributing to disuse osteoporosis. Therefore, it is conceivable that re-introduction of mechanical loading via physical exercise may also reverse atrophy and bone loss in individuals with SCI.

However, though a majority of SCI patients regard physical activity as important, more than half do not have access to appropriate exercise [56]. For the general population, physical exercise is an inexpensive, safe, and effective approach for avoiding health problems. However, a typical individual with SCI experiences many barriers to exercise due to their immobility, such as the inability to use a large portion of their muscle mass, and inability to locate appropriate facilities and affordable equipment. Fortunately, exercise programs based on functional electrical stimulation (FES) have been developed to overcome these barriers. FES-exercise uses electrical stimulation of the paralyzed muscles to cause muscle contractions. Loading the bones through muscular contractions initiated by FES has yielded positive results. For example, in both acute and chronic SCI, up-right standing via force feedback-controlled electrical stimulation of paralyzed quadriceps appears to provide sufficient loading to the paralyzed lower limbs closer to load levels with known osteogenic potential [57]. Moreover, recent adaptation of cycling and rowing exercises for FES provides a new and exciting opportunity to provide mechanical strain to the paralyzed lower limbs sufficient to stimulate bone formation in individuals with SCI.

Recent research has shown that FES-cycling initiated during the very early stages of spinal cord injury (1 – 2 months post-injury) may attenuate the bone loss [58], though at least one study show that this may not be the case [59]. However, the attenuation of bone loss fades quickly, within 6 months once cycling exercise is discontinued [58,60]. Though the reasons for these discrepant results are unknown, one culprit may be the limited mechanical efficiency of cycling exercise. In all individuals, able-bodied or not, exercise must meet certain intensity and volume criteria to induce significant health benefits. For example, passive weight bearing of paralyzed lower extremities appears to be ineffective, and the intensity, frequency, and duration of stress to the bones appear to be important determinants of improved bone parameters [9]. Yet, the mechanical efficiency of FES-cycling is estimated as ~8% [61], less than a third of that for cycling in ablebodied individuals. One issue may be that cycling exercise does not achieve high levels of aerobic work and a plateau in training effect is quickly reached [62]. Therefore, though promising, this modality of FES-exercise may not be sufficient to promote enough bone blood flow and mechanical strain to reliably prevent and reverse SCI-mediated bone loss beyond the very early stages of injury.

In contrast to typical FES cycling exercise, it appears that significant benefits can be achieved via high volume FES cycling training. For example, in patients with chronic SCI, high-volume (five 60-min training sessions a week for 12 months) FES cycling training can partially reverse the loss of bone mineral density [63]. Moreover, though the benefits achieved through 1 year of high volume FES cycling training may be lost if the training discontinues, the benefits appear to be maintained when reduced intensity exercise is continued after the initial training [64]. Recently, in an attempt to overcome the limitations of typical FES-cycling, rowing has been adapted for FES exercise to provide a better exercise modality for individuals with SCI. FES-rowing uses electrical stimulation of the paralyzed quadriceps and hamstrings to actively engage both the arms and the legs in the full rowing cycle. Though it is currently unknown if FES-rowing can prevent osteoporosis during acute phase of SCI, a recent pilot study from our laboratory with three individuals with chronic SCI has shown that the cyclical mechanical loading of the lower extremities during FES-rowing can promote new bone formation (up to 50%), improve bone strength, and may revert osteoporosis during the chronic stage of SCI [65]. Further studies are required to assess the utility of FES-rowing on effective reversal of SCI-mediated osteoporosis. Nevertheless, FES-rowing appears to provide sufficient exercise intensity and mechanical strain to the paralyzed lower limbs to stimulate new bone formation. In addition to the improvement in musculoskeletal health, the advantages of FES-rowing exercise include an improvement in cardiovascular health more than most options currently available [66], the use of a relatively inexpensive ergometer, and integration into existing rowing programs and communities because of its similarity to rowing by the general population. According to participants, FES-rowing is intuitive and easy to learn, and a more engaging and natural exercise, similar to what would be used by able-bodied individuals. Moreover, FES exercise has been shown to be safe for participants [67], and FES-rowing paradigm has been used in our laboratory for exercise by more than 100 individuals with SCI over the past 5 years without any adverse events. Therefore, FES-rowing is offers many new and exciting physiological, economic, and social opportunities for the SCI population.

## Conclusions and Future Directions for Rehabilitative Strategies

Chronic SCI and consequent osteoporosis have a huge impact on the individual, society and the economy, and thus there is need for scientific advances to improve the effectiveness of rehabilitative approaches. In rehabilitation medicine, the shortage of evidence-based practice has been a major barrier to advancing care and promoting the timely identification, application, and assessment of advances in science and technology with the potential to improve rehabilitation outcomes in chronic SCI. For example, sclerostin may provide an alternative therapeutic target during the acute phase of injury as a prevention strategy to prevent initial, rapid osteoporosis, and to improve rehabilitation outcomes in chronic stages. However, future work should define the kinetics of bone loss and its relation to circulating sclerostin when acute mechanical unloading and most bone loss occur. In addition, recent advances in application of a new technology (functional electrical stimulation) provide an exciting new avenue to improve functional mobility, to foster behavioral adaptation to functional losses, and to further facilitate development of improved assistive technologies for individuals with chronic SCI. Future work should address both the physiologic and clinical impact of FES exercise and develop exercise programs that can provide loading to the paralyzed lower limbs sufficient not only to prevent osteoporosis but also to promote osteogenesis to ensure effective reversal of SCI-mediated osteoporosis.
